# Barriers and challenges of implementing pulmonary rehabilitation in Malaysia: Stakeholders’ perspectives

**DOI:** 10.7189/jogh.11.02003

**Published:** 2021-10-30

**Authors:** Soo Chin Chan, Jaspreet Kaur Sekhon, Julia Patrick Engkasan, Jayakayatri Jeevajothi Nathan, Fatim Tahirah Mirza, Su May Liew, Norita Hussein, Anwar Suhaimi, Nik Sherina Hanafi, Yong Kek Pang, Saari Mohamad Yatim, Tracy Jackson, Genevie Fernandes, G M Monsur Habib, Hilary Pinnock, Ee Ming Khoo, RESPIRE Collaborators

**Affiliations:** 1 Department of Rehabilitation Medicine, Faculty of Medicine, University of Malaya, Kuala Lumpur, Malaysia; 2 Department of Primary Care Medicine, Faculty of Medicine, University of Malaya, Kuala Lumpur, Malaysia; 3 Faculty of Health Sciences, Universiti Teknologi MARA, Shah Alam, Selangor, Malaysia; 4 Department of Medicine, Faculty of Medicine, University of Malaya, Kuala Lumpur, Malaysia; 5 Department of Rehabilitation Medicine, Serdang Hospital, Selangor, Malaysia; 6 NIHR Global Health Research Unit on Respiratory Health (RESPIRE), Usher Institute, University of Edinburgh, Edinburgh, UK

Globally, chronic respiratory diseases (CRDs) are one of the most common non-communicable diseases. Of these, asthma and Chronic Obstructive Pulmonary Disease (COPD) are the most prevalent, and post-tuberculosis lung disorders, bronchiectasis and interstitial lung disease are growing problems especially in developing countries [1]. By 2030, CRDs mortality rate is expected to rise to 8.6%, making it the world’s third leading cause of death [2]. Between 1990-2017, the majority of deaths due to CRDs occurred in LMICs, including Malaysia where chronic lower respiratory disease was the 5^th^ leading cause of death in 2018 [3,4]. CRDs cause a significant economic burden to both the patient and society as people living with CRD experience compromised quality of life and reduced work productivity, and frequent hospital admissions incur inpatient care costs [5].

## PULMONARY REHABILITATION

Pulmonary rehabilitation (PR) has proven to benefit patients with CRDs, demonstrating meaningful improvement of symptoms, exercise capacity, mental health, and quality of life [6]. PR also reduces the economic burden due to health costs of patients with CRD, by preventing unnecessary hospital admissions and reducing the length of hospital stays [5]. However, in Malaysia, only one major district hospital provides a structured PR programme consisting of exercise and non-exercise components including: comprehensive assessment tools; education; treatment; breathing techniques; self-management skills such as inhaler techniques, airway clearance and coping strategies. Physiotherapy in most hospitals for patients with CRDs is typically conducted solely by physiotherapists and involves supervised exercise training and chest physiotherapy [7]. A structured multidisciplinary PR programme is lacking due to the scarcity of resources and trained staff [8,9].

Patients with CRDs are referred for physiotherapy by respiratory physicians and there are no standardised guidelines or referral pathways for these patients to receive PR. There is poor awareness among health care professionals about the service provision and referral mechanisms, inadequate rehabilitation services, long waiting lists for PR service, and a perception that patients do not want or need rehabilitation, creating barriers to effective implementation of a PR programme [10]. For patients, transportation difficulties, poor carer support, and inability to commit to schedules of PR programmes [11] result in poor attendance and adherence to PR programmes with dropout rates as high as 50% [12]. A local study carried out in a tertiary referral centre in Kuala Lumpur showed a PR uptake rate of only 16% among 86 patients with moderate-severe COPD and was attributed by the authors to ‘patient refusal’ and ‘low compliance’ [8].

In this paper, we aimed to reflect on the barriers and challenges to the implementation of a PR programme in Malaysia, through the eyes of health care professionals. The intention is to raise awareness of PR’s role in treating patients with CRDs and developing local and national strategies for implementation in the future.

## STAKEHOLDER ENGAGEMENT – PARTNERS IN MEANINGFUL ENGAGEMENT

Stakeholders are individuals, organisations or communities who benefit from having an interest or stake in the outcome of a programme or project [13]. Effective stakeholder engagement requires collaboration, ongoing partnership and shared decision making between the research team and stakeholders to reduce the gap between research evidence and health care decision-making. Stakeholder engagement can be used to constructively implement research findings leading to better patient care, health innovation and policy reform [14]. However, engaging stakeholders in LMICs involves challenges due to ethics, politics, equity, and poverty; therefore, it is essential to address them in a contextual manner.

To maintain a bi-directional relationship, it is important to involve stakeholders throughout every stage of the research to ensure that findings are relevant, beneficial, and ultimately to achieve effective dissemination and implementation [15]. Ideally, therefore, stakeholder engagement typically should commence in the early stages of research to define the research question or identify target populations or aspects of the intervention. Using the 7Ps framework [14], we identified “clinical providers” to be the primary stakeholder as they are closely integrated in shared decision making with patients. Specifically, these were identified to be those most closely involved in the care of patients with CRDs including physicians, therapists, nurses, and medical assistants. However, long-term aims are to include other stakeholders such as patients and the public, policy makers, and purchasers for further PR implementation research.

We conducted a virtual stakeholder engagement event using the Zoom platform that consisted of several information sessions by various health care professionals with relevant expertise in PR and discussions in breakout sessions to raise awareness of PR and explore perspectives on implementing a PR programme in Malaysia. Invitations to the event were sent to all hospital directors and heads of departments in public and private hospitals with rehabilitation services with a request to involve the multidisciplinary team including allied health personnel such as physiotherapists, occupational therapists, and medical assistants. The barriers identified and proposed solutions were based on feedback given by stakeholders on their individual experiences within their health care institutions. The National Institute of Health Research (NIHR) recommends undertaking stakeholder engagement to meet the needs and priorities of end users of research, however these activities are not commonplace in Malaysia, and this is one of the first Stakeholder Engagement activities for CRDs with regards to PR.

## BARRIERS AND CHALLENGES OF IMPLEMENTATION OF PULMONARY REHABILITATION (PR) PROGRAMME IN MALAYSIA

In April 2021, we were joined by 110 health care professionals from public and private hospitals across every state in Malaysia. Almost all (98%) attendees were interested in initiating a PR service in their institution with a preference for delivering home-based PR programmes (61%). The most significant barrier identified to PR programme implementation in Malaysian health care institutions was a lack of knowledge on PR among health care professionals which deterred initiation of programmes at their institutions due in part to a lack of formal training to deliver PR effectively.

There appeared to be a lack of personnel to conduct and deliver an effective PR programme due to the limited time, manpower and resources at all levels of health care institutions in Malaysia, from primary health care clinics to tertiary referral centres. In addition, expansive hospital institutions posed practical issues such as needing to walk lengthy distances between multiple departments involved in delivering effective respiratory health care, which is exceptionally difficult for patients as it exacerbates breathlessness, one of the main distressing symptoms of CRD. Furthermore, there are structural limitations to hospital environments, for example, insufficient space to carry out baseline PR activities or assessments such as the 6-minute walking test (6MWT) which requires a 30-m straight walking track.

Stakeholders highlighted the lack of a structured PR programme or guidelines for care and referral. In certain institutions, rehabilitation for patients with CRDs consisted of an outpatient exercise programme only. The lack of a structured programme coupled with a lack of training resulted in many health care professionals not having the confidence to deliver PR, especially to patients requiring oxygen therapy.

Some patients may be too ill to enrol in a PR programme or have comorbidities that prevent them from fulfilling the exercise requirements of the programme. For example, patients on long-term oxygen therapy or in severe stages of their illness; patients who cannot commit to weekly PR sessions; patients are not motivated to carry out PR, potentially due to their lack of knowledge and awareness about the importance of PR in improving their lung function and overall health. Some patients who live in rural and remote areas could have problems accessing PR services as it is not readily available in all hospitals, especially district hospitals. One attendee suggested holding an outreach programme, which could be difficult to implement currently due to the COVID-19 pandemic. While government subsidies aid in the direct costs of PR sessions, the indirect costs, such as travel or salary loss, may be too much for patients to bear.

Key facilitators to a successful PR programme were highlighted including having a proactive and knowledgeable programme director; dedicated and motivated programme coordinators; committed staff of highly skilled professionals who specialise in practicing a transdisciplinary approach to conducting PR. Notably, including peer support groups in the programme was highlighted as essential to foster communication and support.

**Table 1** summarises the barriers and solutions to implementation of PR programmes in Malaysia from the stakeholder engagement event.

**Table 1:** A summary of the barriers and solutions to implementation of PR programmes in Malaysia

Barriers to implementation of PR in Malaysia	Proposed solution
Low knowledge and awareness of PR among health care professionals	Formal PR training of health care professionals; appointment of motivated programme coordinators with expert knowledge of PR
Human resources, logistics and accessibility	Installation of appropriate facilities and smooth communication pathways between departments in hospitals; utilising existing resources of health care professionals to carry out PR
Lack of a structured PR programme	Provide written guidelines, protocols and streamlined referral pathways
Patient barriers to participation	Community outreach programmes with hospital-community collaboration; patient education and awareness about PR; health care professionals working with patients to understand their needs and expectations regarding PR

PR – pulmonary rehabilitation

## IMPLICATIONS FOR RESEARCH

The key factors of limited awareness; poor multidisciplinary involvement; a lack of referral pathways and proper facilities have significant implications for future research plans that aim to measure the effectiveness of PR.

It is important that people living with a CRD be provided sufficient information and education of their disease and mechanisms by which improvements can be achieved. This will allow researchers to identify important measurable outcomes that may reflect meaningful benefit for patients. Concurrently, health care professionals need to understand patients’ expectations and needs to deliver patient-centred holistic care. To foster PR awareness in the community, hospital-community collaborations should be developed to identify rural and remote community needs and improve the delivery of PR through centre- or home-based programmes.

Health care professionals require formal training in PR, particularly from institutions with an established programme with trainers that possess the expertise in implementing PR. It is important for hospitals to build/adapt appropriate facilities for PR and to have smooth communication pathways between departments. Alternatively, health care professionals can adopt available spaces to carry out PR, for example, using empty hospital corridors for 6MWT or use other validated tools which needs less space eg, Incremental Shuttle Walking Test (ISWT) or 6-Minute Stepper Test (6-MST). Development of evidence-based guidelines and clinical referral pathways for PR will enable better patient care and recruitment for future research.

## LIMITATIONS AND STRENGTHS IN THE STAKEHOLDER ENGAGEMENT MEETING

Due to the COVID-19 pandemic, this meeting was conducted virtually. The remote format allowed stakeholders from geographically widespread communities to participate with no travel time. However, some of the stakeholders were unable to contribute due to poor network connectivity. Main decision makers such as the hospital directors, national heads of the rehabilitation services, Ministry of Health strategic planning and development representatives who play a high influencing and strategic role to the implementation of PR were unable to attend due to other clinical responsibilities.

## CONCLUSION

The stakeholder engagement activity for PR implementation has enabled an understanding of the needs and priorities of stakeholders and helped us identify solutions for barriers for research on PR implementation.
